# Novel Hybrid Polymer Composites Based on Anthraquinone and Eco-Friendly Dyes with Potential for Use in Intelligent Packaging Materials

**DOI:** 10.3390/ijms222212524

**Published:** 2021-11-20

**Authors:** Anna Masek, Angelika Plota, Julia Chrzastowska, Małgorzata Piotrowska

**Affiliations:** 1 Faculty of Chemistry, Institute of Polymer and Dye Technology, Lodz University of Technology, Stefanowskiego 16, 90-537 Lodz, Poland; angelika.plota@dokt.p.lodz.pl (A.P.); julia.chrzastowska@gmail.com (J.C.); 2 Faculty of Biotechnology and Food Sciences, Institute of Fermentation Technology and Microbiology, Lodz University of Technology, Wolczanska 171/173, 90-530 Lodz, Poland; malgorzata.piotrowska@p.lodz.pl

**Keywords:** polymer composites, aging indicators, weathering, thermo-oxidation, quercetin, smart packaging

## Abstract

This study aimed to present the influence of bio-based and anthraquinone dyes and their combinations on the optical properties of ethylene-propylene (EPM) composites after thermo-oxidative and climatic aging. Therefore, the chosen polymer was filled with a natural, plant-origin flavonoid—quercetin, and with two commercial anthraquinone dyes (C.I. Solvent Yellow 163 and C.I. Solvent Red 207). The manufactured polymer composites were subjected to accelerated aging tests: weathering and thermo-oxidation, respectively. Examination of the materials’ properties indicated that the combination of synthetic and natural dyes can result in better resistance to oxidizing agents and higher thermal stability of ethylene-propylene products. Moreover, color change of quercetin-containing samples due to exposure to simulated atmospheric conditions could be a promising solution for use as aging indicators in intelligent packaging materials that will inform about the ongoing degradation process. Another interesting finding is that these samples exhibited good fungistatic activity against *Candida albicans* yeast and *Aspergillus niger* mold. Overall, this novel solution based on hybrid polymer composites containing natural and commercial dyes is a more environmentally friendly alternative to traditional materials used in the plastic packaging industry with better and more desirable properties.

## 1. Introduction

In the available literature, a huge number of well-known chemical additives that are added to the polymer matrix can be found. Most often they improve processability, extend service life, and/or allow the desired chemical and physical properties of high-quality finished polymer products to be obtained. The definition of an additive in accord with the European Community is as follows: “a substance, which is incorporated into plastics to achieve a technical effect in the finished product, and is intended to be an essential part of the finished article” [1,2,3]. Typically, their content in the polymer matrix is only a few percent but this is sufficient to ensure the material stability and meet the requirements of varied applications [4,5].

Dyes and pigments constitute a wide group of polymer additives that are mainly used to make the product attractive for the consumer by its diverse coloration. However, they can also have a significant influence on parameters such as chemical resistance, thermal stability, durability, or resistance to various environmental factors, including elevated temperature, ultraviolet (UV) radiation, and humidity [6,7,8,9,10]. For instance, Cavalcanti et al. (2019) [11] examined the influence of irgalite red 2BSP pigment on the photodegradation of polypropylene (PP) films. They observed that the presence of this pigment improved the ultraviolet stability when evaluated by UV-Visible and infrared spectroscopy. A significant difference between a dye and a pigment is their solubility. Dyes are chemical substances that must be soluble in the application medium (e.g., water) whereas pigments are compounds composed of particles, which are insoluble in the used medium. Both dyes and pigments are widely applied in areas such as: cosmetics, textiles, food, packaging materials, pharmaceutical, paper, and in many other industries, where they should show good compatibility with the colored product, high color intensity, and low tendency to migration [12].

Commercial colorants used in this study are two solvent dyes, which in terms of their chemical structure belong to the group of anthraquinone dyes. This group of dyestuffs is a relatively novel solution in the dyeing industry, and is characterized by light and chemical resistance, great thermal stability under processing, and good solubility in non-polar liquids. Unlike traditional dyes, they are basically not water-soluble. Furthermore, the great advantage for the customer is the fact that they are inexpensive and have a high color intensity. In general, they are used in products in which they dissolve, such as waxes, fats, polymers, or varnishes [13,14,15].

On the other hand, considering the impact on the environment and the growing constraints on the composition of, for example, packaging materials, synthetic substances are increasingly being replaced with natural compounds [16,17,18,19,20]. For instance, the textile industry produces and employs approximately 1.3 million tons of synthetic dyes and pigments all over the world, which cost about $23 billion. Unfortunately, they can generate hazardous chemical substances which affect the natural environment and human health [21]. Therefore, more and more often, plant-origin compounds are used as color additives that are an attractive alternative to traditional dyes and meet the national and international restrictive requirements [22]. In general, natural dyes are extracted from various fruits, vegetables, or fungal species, and they are commonly used in such areas as medicine, textiles, food coloration [23], packaging materials [22], leather processing [24], or handicraft products [25]. Furthermore, these natural substances very often are characterized by antibacterial and antifungal action, antioxidant properties, and anti-carcinogenic effect. One example of such compounds may be quercetin, which can be obtained from hawthorn and chestnut flowers, onion, dark grape, or black cumin seeds. In the available literature, it is characterized not only as a coloring agent (brown-red, orange, and olive-black colors) but also as powerful antioxidant with anti-carcinogenic, anti-inflammatory, and antiviral properties [26]. As a result of its antioxidant properties, quercetin has the ability to scavenge free radicals, thus preventing a premature degradation of the polymer products [27].

Nowadays, intelligent packaging solutions are the subject of many scientists’ research. Constantly growing consumer expectations to have high-quality food products have led to the emergence of new technological solutions in the plastic packaging industry that inform the consumer about the condition of the stored food. Some studies on constructing and using smart packaging have been carried out. Zhang et al. (2019) [28] developed smart sensors based on anthraquinone dyes with pH-sensitive azo chromophores by printing patterns that change the color of paper packaging. Their results showed timely and sensitive color change from green to purple and red to green for two synthesized compounds, in what proved to be a good potential application in food freshness identification. Dirpan et al. (2018) [29] utilized a bacterial cellulose from Acetobakter xilinum, which was then soaked with bromophenol blue solution and obtained a freshness indicator for mango fruit. In their research, the color change from dark blue indicating fresh fruit, light blue—firm, and green color indicating the fruit is rotten, was observed. Moreover, Luzi et al. (2019) [30] added quercetin as an active ingredient to poly(vinyl alcohol) films intended for food packaging. Their results confirmed that this polymer in combination with quercetin can be successfully used to produce smart packaging, as a deviation in the red color of the material was observed after 7 and 21 days of exposure under simulated conditions. The huge potential of quercetin as a substance applied in the intelligent packaging systems was also described by Łopusiewicz et al. (2021) [31], who examined the influence of this compound on the bioactive poly(butylene-succinate) films. Their research showed significant changes in coloration, UV blocking effect and, importantly, the prepared composites were characterized by absolute free radicals scavenging and antibacterial properties. Another interesting example of color changing technology is a color changing coffee cup lid developed by Smart Lid Systems (Sydney, Australia). This modern solution is based on the fact that the mug lid impregnated with a special color-changing additive informs the consumer that the drink is not too hot.

In the following study, a novel hybrid solution based on anthraquinone and eco-friendly dyes and their combinations introduced into an ethylene-propylene matrix (EPM) is proposed. The manufactured samples were subjected to weathering and thermo-oxidation processes, which lasted 200 and 400 h, respectively, and the effect of selected additives on the optical properties of EPM samples after accelerated aging tests has been investigated. The combination of quercetin with solvent dyes resulted not only in better resistance to oxidizing agents and higher thermal stability of EPM composites, but it also increased the ecological profile of the manufactured polymer products.

## 2. Results and Discussion

### 2.1. Characterization of Solvent Dyes and Quercetin Powders

**Figure 1:**
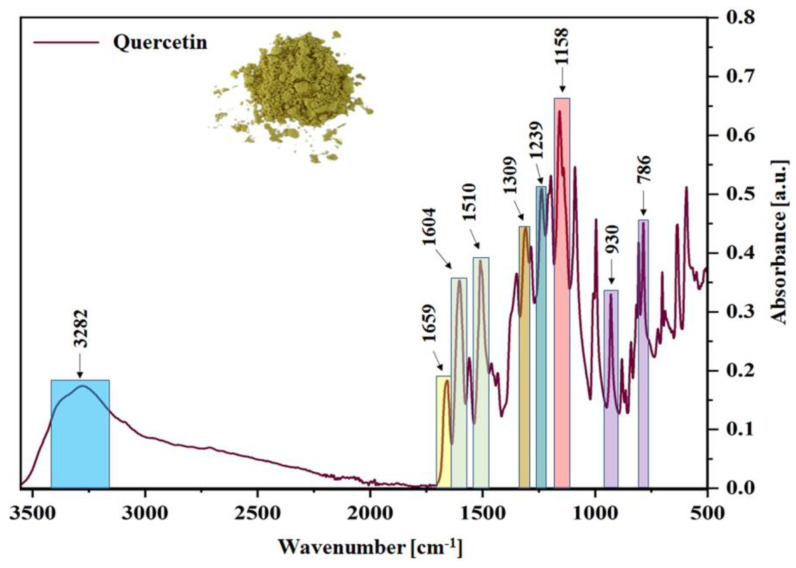
FT−IR absorption spectrum of pure quercetin powder with its characteristic peaks marked.

**Figure 2:**
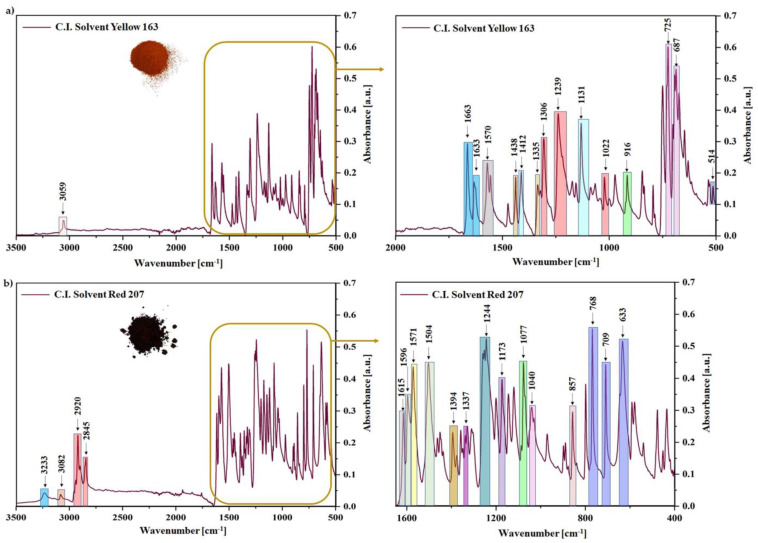
Fourier-transform infrared spectroscopy (FT−IR) spectra of (**a**) C.I. Solvent Yellow 163 and (**b**) C.I. Solvent Red 207 recorded in the range of 400−4000 cm^−1^.

**Figure 3:**
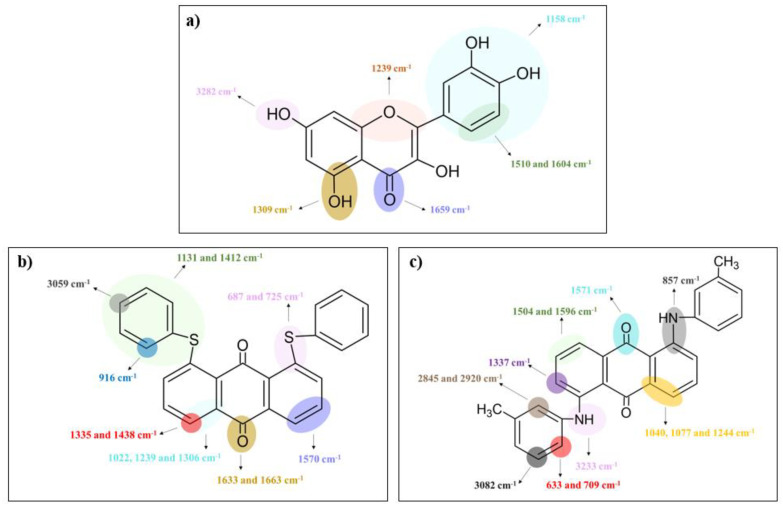
Absorption bands assigned to the chemical groups (bonds) that occur in the powders of (**a**) quercetin, (**b**) C.I. Solvent Yellow 163, and (**c**) C.I. Solvent Red 207.

Fourier-transform infrared (FT-IR) spectroscopy was carried out to define the specific functional groups of quercetin and solvent dyes (C.I. Solvent Yellow 163 and C.I. Solvent Red 207), with which the radiation was interacting. This research made it possible to better understand the effect of the dyes’ structure on the aging behavior of ethylene-propylene rubber, which is a key aspect in terms of the service life of a given polymeric product. Figure 1 and Figure 2a,b show the obtained spectra for all tested compounds, where their characteristic bands were detected and assigned to the specific functional groups (bonds) in Figure 3.

Figure 1 presents the FT-IR spectrum for pure quercetin powder, one should notice some characteristic absorption bands, e.g., a broad band of hydroxyl groups stretching at 3282 cm^−1^, whereas the stretching associated with =C-OH of the phenol group was recorded at 1309 cm^−1^. Moreover, carbonyl group (C=O) was observed at 1659 cm^−1^ and characteristic bands of C=C bonds stretching were detected at 1510 and 1604 cm^−1^. The FT-IR spectrum for C.I. Solvent Yellow 163 powder is presented in Figure 2a. In this case, the characteristic band related to the ring-sulphur stretching was observed at 1131 and 1412 cm^−1^, bands at 1022, 1239, and 1306 cm^−1^ were assigned to the C-C stretching vibrations, whereas at 1633 and 1663 cm^−1^ to carbonyl groups stretching (Figure 3). However, the second synthetic colorant—C.I. Solvent Red 207 was characterized by N-H bond stretching vibrations at 3233 cm^−1^ (Figure 2b and Figure 3). Bands at 1504 and 1596 cm^−1^ were attributed to the C=C stretching and at 1571 cm^−1^ to the carbonyl bond stretching. The above assignments are in good agreement with the literature values [32,33,34,35,36,37,38,39,40,41,42,43,44,45,46]. More detailed analysis of all specific functional groups presented in the tested samples is shown in Figure 3.

**Figure 4:**
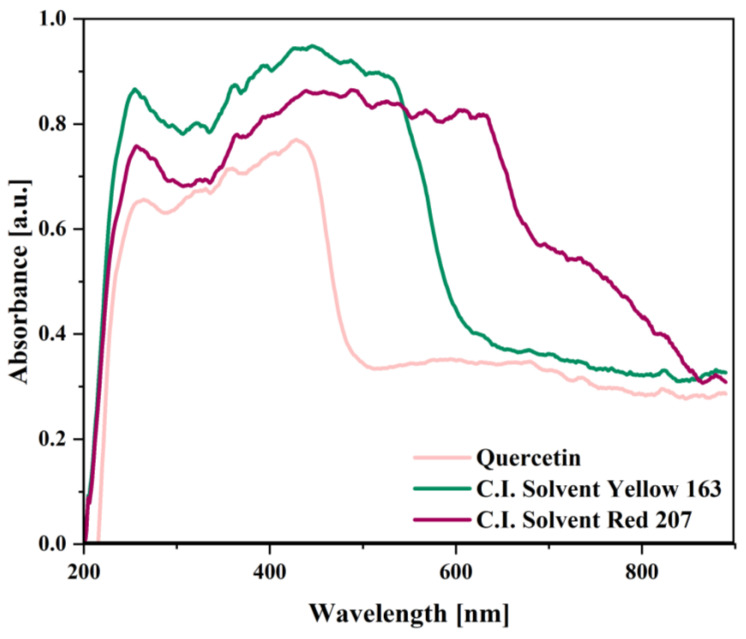
UV-Vis spectra recorded in the range of 190–1100 nm obtained for the tested natural and synthetic dyes: quercetin, C.I. Solvent Yellow 163, and C.I. Solvent Red 207.

In addition to FT-IR analysis, the optical properties of quercetin and two commercial solvent dyes—C.I. Solvent Yellow 163 (S.Y. 163) and C.I. Solvent Red 207 (S.R. 207) were investigated by UV-Vis spectroscopy in the range of 190 to 1100 nm. The obtained absorption spectra for the tested colorants are presented in Figure 4 and are characterized by two specific peaks. The first of them reached a maximum at 250 nm for all samples, while the second one saw a broad peak between 290 and 420 nm for quercetin, between 340 and 540 nm for S.Y. 163, and between 340 and 630 nm for S.R. 207.

Quercetin belongs to the flavone aglycones group, which is a subgroup of flavonoids. In general, the UV-Vis spectra of flavones and related glycosides feature two absorption peaks corresponding to band I (300–380 nm), which is attributed to the B-ring (cinnamoyl system) and band II (240–280 nm), which is associated with the A–C benzoyl system [47,48]. Moreover, substituents presented on the A and/or B ring may be responsible for producing bathochromic and hyperchromic absorption shifts. However, in the case of both anthraquinone dyes used in this study, the second peak was much broader than for quercetin and the maximum recorded for S.Y. 163 was 445 nm and for S.R. 207 was 487 nm. Anouar et al. (2014) [49] adverted that the various shades of anthraquinone colorants are dependent on the positions of auxochromic groups presented on their skeleton and their UV-Vis spectra show absorption bands between the range of 220–350 nm (e.g., π→π* electronic transitions) and also one broad band that is close to 400 nm. By analyzing the UV-Visible spectra of anthraquinone dyes that are given in Figure 4, the influence of substituents pattern on the sample color can be observed. The introduction of electron donating groups into the anthraquinone solvent dyes contributes to bathochromic shift, which is dependent on their position, number, and relative strength. Moreover, as it is well known, electron donating groups exhibit stronger bathochromic interactions in α-positions (1, 4, 5, 8) than in β-positions (2, 3, 6, 7) [14]. In this study, the effect of hyperchromic shift for S.Y. 163 was noted by its greater absorbance in comparison to S.R. 207. On the other hand, a bathochromic shift ($\lambda_{\max}=487\;\mathrm{nm}$) was observed for S.R. 207 dye, which can be caused by the amino group existing in its structure confirmed by the FT-IR study.

**Figure 5:**
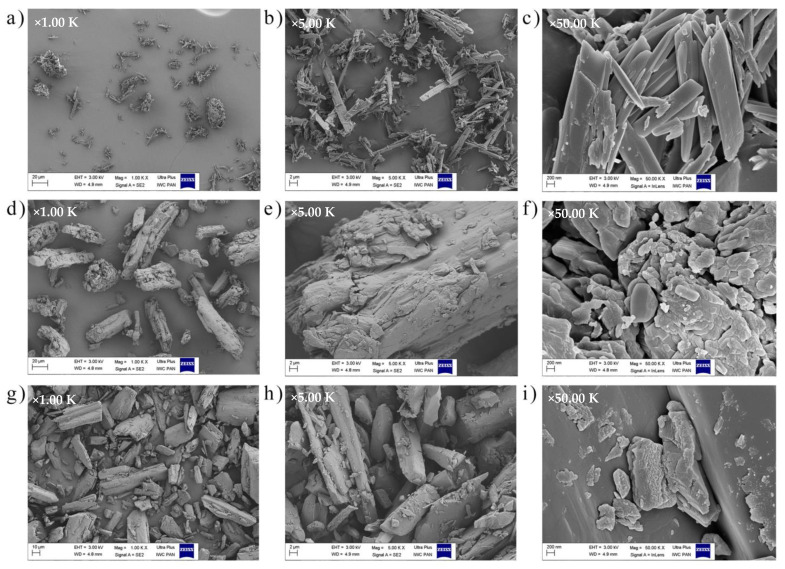
Scanning electron microscopy (SEM) images obtained for (**a**–**c**) quercetin, (**d**–**f**) C.I. Solvent Yellow 163, and (**g**–**i**) C.I. Solvent Red 207 powders at a 1000, 5000, and 50,000× magnification, respectively.

The structure of dye crystals is often examined by scanning electron microscopy (SEM). In general, dye particles have a crystalline structure, but lose their crystallinity during processing and form a molecular solution in the colored medium. Figure 5 presents SEM images obtained for colorant powders at different magnifications, 1000, 5000, and 50,000×, respectively. Usually, dye molecules occur in the form of conglomerates, in which respective particles can be identified. On the basis of SEM images, different shapes and sizes of particles in the used compounds can be seen and, as shown in the literature, these factors may influence the physico-chemical properties of substances, e.g., their optical properties [50]. The quercetin morphology was characterized by strip-like structure and the surface of its granules seemed smooth. Length range of these crystalline particles is from 2 to 24 μm and their tendency to aggregate and agglomerate is clearly visible. A similar effect was observed by Lv et al. (2019) [51]. However, in the case of Solvent Yellow 163 crystalline particles, there is an apparent irregular shape and morphology of the crystallites itself. It seems that smaller rod-shaped parts of crystallites are attached to the bigger ones, forming the uneven surface. Moreover, their length is in the range of approximately 200 nm to 40 μm. The second synthetic colorant—Solvent Red 207 was characterized by brick-shaped crystallites with visible sharp edges. The tendency to forming aggregates and agglomerates is the lowest in comparison to quercetin and Solvent Yellow 163 powders, however, crystallites clusters are still evident. In this case, particle size is approximately from 2 to 80 μm.

### 2.2. Characterization of Ethylene-Propylene Composites

#### 2.2.1. Morphology and Optical Properties

**Figure 6:**
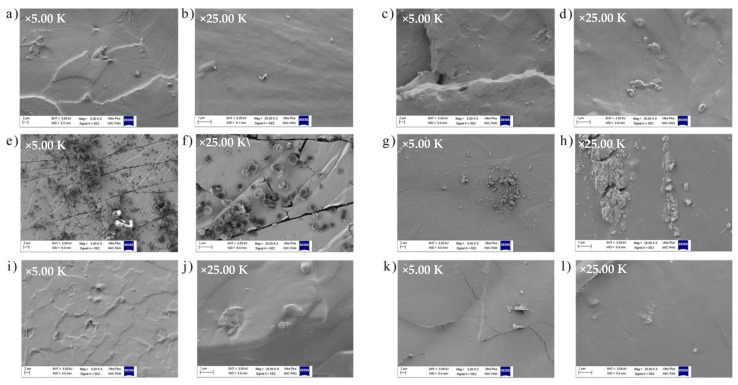
SEM images obtained for the following materials: (**a**,**b**) pure ethylene-propylene rubber (EPM), (**c**,**d**) EPM-quercetin, (**e**,**f**) EPM-S.Y. 163, (**g**,**h**) EPM-S.R. 207, (**i**,**j**) EPM-quercetin-S.Y. 163, (**k**,**l**) EPM-quercetin-S.R. 207 at a 5000 and 25,000× magnification, respectively.

A distribution of the dye particles in the polymer matrix plays a key role in determining its optical properties and may also affect the resistance of polymer composites to UV radiation. Figure 6 shows the SEM images of ethylene-propylene materials with quercetin, S.Y. 163 and S.R. 207 dyes before aging processes.

**Figure 7:**
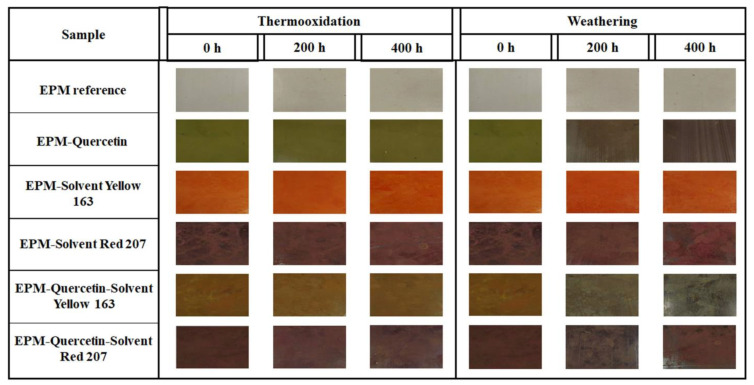
Visualization of ethylene-propylene based materials before and after 200 and 400 h of thermo-oxidation and weathering.

In general, solvent dyes are non-ionic colorants, which show no solubility in water but, on the other hand, they are soluble in various types of solvents. Moreover, they are used for dyeing materials in which they can dissolve, e.g., plastics, fats, waxes, or inks. They usually have very good dispersion due to the fact that during processing they lose their crystallinity and form a molecular solution in the dyed medium. But considering the SEM images presented in Figure 6, especially in the case of EPM rubber with C.I. Solvent Yellow 163 and C.I. Solvent Red 207 content, particle agglomerates can be seen. In addition, microstructure of ethylene-propylene with S.Y. 163 content was characterized by numerous cracks. However, for samples, where synthetic dye was combined with natural—quercetin, this effect was not so visible. Another undesirable feature noticed in composites filled with anthraquinone dyes (particularly with S.R. 207—magenta) was their migration to the polymer surface, which is easily visible in Figure 7 (rows 4 and 6). This phenomenon is called the dye blooming effect, whereby dye crystals are formed on the film surface during the cooling process and within 1 day of storage at room temperature. Therefore, in order to avoid the dye’s crystallization and its migration to the surface, which may in fact be of practical importance, e.g., in packaging or coating applications [52], Hariri et al. (2009) [53] proposed the use of block copolymers that are able to selectively solubilize a colorant powder. It can also be assumed that solvent dyes’ efflorescence appeared because of interaction between the dye functional groups and the cross-linking system. Therefore, these kinds of dyeing substances could be better suited to polymeric materials that do not need to be cross-linked using cross-linking system, e.g., some types of thermoplastics, thermoplastic elastomers, or copolymers.

The next part of the research involved colorimetric measurements taken before and after 200 and 400 h of the simulated aging processes. The coloration of the polymer composite is provided by dyes, as a result of the selective absorption of visible light in the wavelength range from 380 (violet) to 760 (red) nm. The main element of the dye structure, responsible for light absorption, is the chromophore group, e.g., -C=C-, -C=O-, -NO_2_. Moreover, dyes very often also contain electron donating or electron withdrawing substituents (auxochromes), which can affect the color intensity of the chromophores. For instance, dyeing compounds based on the 9,10-anthraquinone structure are slightly colored, but their appearance can be changed by the introduction of electron donating groups such as -OH or -NH_2_, which leads to a bathochromic shift and better color strength.

**Figure 8:**
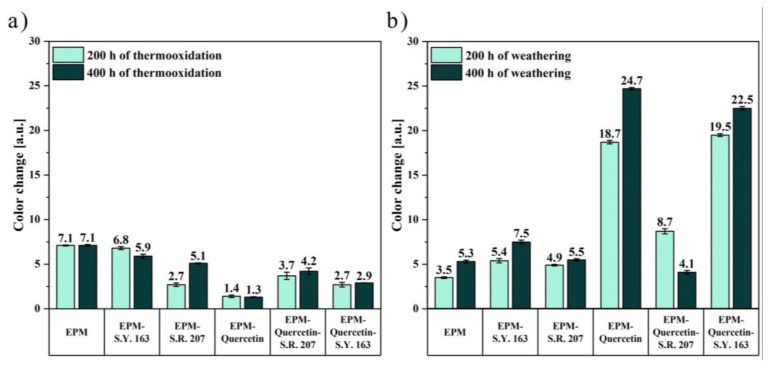
Total color difference (ΔE) of EPM composites subjected to (**a**) thermo-oxidation and (**b**) weathering for 200 and 400 h, respectively.

The effect of thermo-oxidative aging and weathering on the color change of samples can be observed in Figure 7 and Figure 8. As is well known, various environmental factors such as elevated temperature, ultraviolet (UV) radiation, and humidity can alter the physico-chemical properties of polymer products. Flavonoids, including quercetin, have a wide range of pigmentation, but are most common from yellow to red/purple. All of them own a hydroxyl group (OH) in the R-4′ position, including the red-violet (magenta) cyanidin-based pigment [54]. Importantly, they can absorb UV radiation and thus provide protection against damaging UVA and UVB radiation. Their attractive property is color change depending on various environmental factors. In Figure 7, the most characteristic color change under the influence of climatic aging was observed for the ethylene-propylene composites containing quercetin (mainly for samples placed in rows 2 and 5). The EPM-quercetin specimen was characterized by a green (artichoke) color before aging, but after prolonged exposure to the climate space, it turned bark root brown (the color of black chocolate) after 400 h. However, in the case of EPM-quercetin-S.Y. 163 composite, as a result of weathering, a color change from fulvous to greenish-brownish shade was noticed. Only when the EPM rubber contained a combination of quercetin with a synthetic dye—S.R. 207 (magenta), the color change was much smaller and a slight discoloration of the material was observed. The reason may be the pigmentation of the C.I. Solvent Red 207 dye (eggplant shade), which is similar to that obtained with quercetin-containing samples after aging processes. It should also be emphasized that the smallest coloration changes were found under the influence of the thermo-oxidation process, and thus it can be assumed that the quercetin stabilized ethylene-propylene polymer is resistant to this environmental factor.

Colorimetric measurements made it possible to examine differences in an EPM surface’s color and assess whether the flavonoid alone and in combination with solvent dyes can act as natural aging time indicator, which would be a great advantage and a possibility for use in intelligent packaging materials. Figure 8 shows the total color difference (ΔE) of the EPM-based samples, which was calculated from the visible spectra in the CIE-Lab space. As observed above in Figure 7, also the ΔE values obtained for the materials subjected to the climatic aging were much higher, especially for composites containing quercetin. Therefore, the correlation between the visual assessment and the results obtained from spectrophotometric tests is very good.

In the case of ethylene-propylene rubber, the color change (ΔE) was equalled to 7.1 after 200 and 400 h of thermo-oxidation and nearly 5 after climatic aging. This polymer without additives is transparent, hence the color differences in this case are small, and the fact that it clearly turned yellow is not a desirable property of polymer products. On the other hand, samples with the addition of various dyes subjected to thermo-oxidative aging were characterized by better color stability, which may mean their higher resistance to increased temperature. The greatest color change was observed for EPM materials containing quercetin. After 200 h of weathering, this parameter was approximately 20 for EPM-quercetin and EPM-quercetin-S.Y. 163 samples and over 8 for EPM-quercetin-S.R. 207 composite (Figure 7 and Figure 8b). All the above-mentioned samples had different shades of dark brown color after 400 h of exposure to simulated atmospheric conditions. The change in color of quercetin-containing polymeric materials caused by weathering may depend on the structure of this natural flavonoid, which oxidizes under the influence of climate space and thus changes its color, constantly protecting the polymer matrix. According to the literature data, flavonoids show strong antioxidant properties, because they react easily with free radicals, acting as free radical scavengers and oxidize at the same time. One of them is quercetin, the polyphenolic structure of which containing numerous double bonds and hydroxyl (OH) groups confirmed by FT-IR test, enables the donation of electrons to stabilize free radicals. The possible mechanism of free radical scavenging and quercetin oxidation has been extensively described, for example, by Fuentes et al. (2017) [55]. Two different quercetin pharmacophores, the catechol moiety in ring B and the 3 hydroxyl (OH) groups in rings A and C, influence its antioxidant properties, with the activity of groups in rings A and C being enhanced by an electron donating effect of the hydroxyl groups located at 5 and 7 positions. Furthermore, the OH groups in the B and C rings are able to stabilize the active intermediates, and the C-3 hydroxyl group has this capability because it can form intermolecular hydrogen bonds with oxygen at the C-4 position. Therefore, the addition of quercetin not only prevents or delays degradation of the polymer matrix, but also changes color as a result of its oxidation, which can be considered an advantage as such materials would be excellent aging indicators when used in smart packaging materials. For instance, Singh et al. (2018) [56] described a possibility of using anthocyanins in intelligent food packaging systems that have the potential to act as pH-sensing films and provide information on the quality of food products. Importantly, such technologies should be non-toxic, sensitive, cheap and stable.

In addition, Table 1 shows the differences in the yellowness index (L*) and two a* and b* coordinates according to the CIE-Lab system, which were obtained on the basis of spectrophotometric measurements. The L* index is related to the level of light or darkness and its values range from 0 to 100. The a* index is a red-green coordinate, where a positive value of a* means red color and negative means green. On the other hand, the b* index is a yellow-blue coordinate, for which a positive value indicates yellow color and negative—blue.

The same tendency as for color change (ΔE) was observed for the obtained results of L*, a* and b* parameters, which were characterized by greater differences in values caused by weathering in the samples containing quercetin. Comparing the L* index values for all tested materials before aging, the pure EPM sample was the brightest (L* = 90.6), and the darkest was EPM-S.R. 207 composite (L* = 26.5), which is consistent with Figure 7. Moving forward, after 400 h of climatic aging, EPM-quercetin and EPM-quercetin-S.Y. 163 materials had a yellowness index above 30, which was very close to the value obtained for the EPM-S.R. 207 sample (L* = 27.6). As described above, films with quercetin content changed color towards a dark brown shade with a progressive degradation of the material, which resembled the color of the sample with the addition of C.I. Solvent Red 207 dye. Therefore, the values of the L* index for these composites were similar. According to the results of a* and b* parameters, the a* value for ethylene-propylene rubber with quercetin changed significantly towards red, and the b* coordinate—towards blue. Based on these findings, it can be assumed that the green chromophores presented in this sample were degraded under the influence of simulated atmospheric conditions, and thus the a* coordinate changed its hue towards red. A similar trend was observed for the EPM-quercetin-S.Y. 163 composite, whose yellow chromophores had lost their properties, and the b* coordinate showed a color shift towards blue. All these changes observed during spectrophotometric tests are related to the chemical structure and properties of quercetin. During the aging processes, under the influence of various atmospheric factors, it reacts with free radicals, oxidizes and thus protects the polymer matrix [57]. It has been clearly proven in the literature that the B-ring in its structure is the main H-transfer site and thus is of the greatest importance for the antioxidant activity [58]. In this study, during FT-IR analysis (Figure 1), the catechol moiety was B-ring. In addition, the 2,3-double bonds present in A-ring also have an effect on the antioxidant capacity of quercetin as they contribute to the π-electrons delocalization and thus are responsible for ${\mathrm{RO}}^{.}$ stabilization. However, the opening of the C-ring promotes the formation of phenolic acids. Therefore, the chemical structure of this compound is of great importance for the aging behavior of the EPM films. The color change of ethylene-propylene rubber with the dyes tested, calculated on the basis of the L*, a*, b* parameters, results from the oxidation process of quercetin.

**Figure 9:**
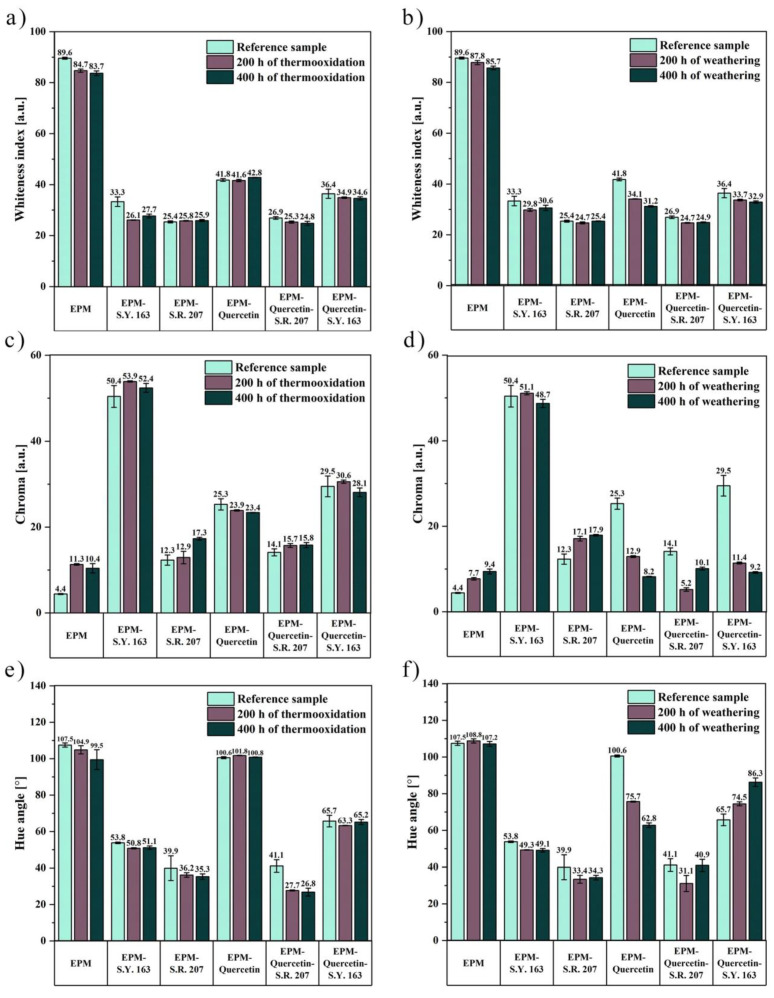
Additional parameters describing the color change of the tested samples, (**a**,**b**) whiteness index, (**c**,**d**) chroma, (**e**,**f**) hue angle, before and after thermo-oxidative and climatic aging, respectively.

Moving forward, based on the determined values of the yellowness index (L*) and two coordinates (a* and b*), more data in relation to the color change, namely the whiteness index, chroma and hue angle calculated from Equations (6)–(8) are shown in Figure 9.

The differences in the values of the whiteness index shown in Figure 9a,b were practically imperceptible both after thermo-oxidation and weathering. Only in the case of ethylene-propylene sample with quercetin content subjected to climatic aging, its darkening was observed (WI = 41.8 before aging and 31.2 after 400 h of weathering). Another color parameter analyzed in this study was chroma, which informs about the colorfulness of the sample. It should be emphasized that the higher the chroma values, the greater the color intensity of the tested products. The changes obtained in the chroma values shown in Figure 9c,d were much more visible, especially under the influence of atmospheric conditions. The chroma values for samples with quercetin addition are much higher compared to pure ethylene-propylene rubber before aging. This fact is not surprising as it is related to the yellow color of quercetin powder, which is attributed to conjugation in the B-ring cinnamoyl system [59]. By analyzing the chroma of EPM composites after weathering, pure EPM and EPM rubber with the addition of C.I. Solvent Red 207 dye showed an increase in the degree of their saturation after 400 h. On the other hand, for other materials, a decrease in these values was observed, which may indicate a discoloration of the material, i.e., increased transparency, and it can be caused by a reduction in the amount of crystal structures in the tested sample. Notably, the largest change in the chroma value was observed for EPM-quercetin-S.Y. 163 film. Figure 9e,f presents the hue angle (Δh) differences obtained for ethylene-propylene materials. In general, the hue angle corresponds to the circle of shades and ranges from 0 to 360 degrees. As mentioned earlier, the a* index is the redness-greenness coordinate and the b* is the yellowness-blueness component, thus h = 0° resembles red color, h = 90° yellow, h = 180° green, and h = 270° blue of polymer surfaces. Take these relationships into account, the hue angle for all materials varied from 0 to 110° on the shadow circle, and their tone was between yellows and reds. The greatest change in this parameter caused by climatic aging was observed for the EPM-quercetin composite, for which a significant decrease in the hue angle value was obtained, which means that its shade became more red.

To summarize the optical properties of the tested ethylene-propylene materials, the highest color stability was observed for composites containing only a synthetic dye (C.I. Solvent Yellow 163 and C.I. Solvent Red 207). On the other hand, the addition of quercetin to EPM systems considerably influences the color of the tested samples. This fact confirms that it can not only increase the ecological potential of a polymer product but can also be successfully used alone or in combination with synthetic dyestuffs in bio-based intelligent packaging materials that will inform about the ongoing degradation process.

#### 2.2.2. Surface Properties

**Figure 10:**
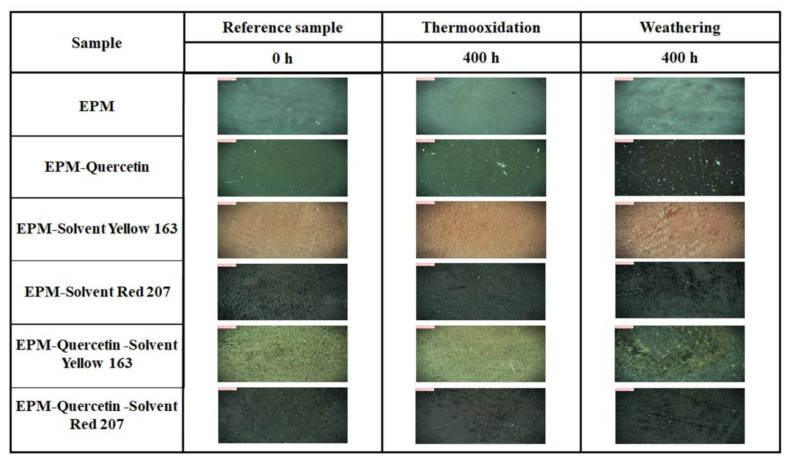
Effect of thermo-oxidation and weathering aging on the surface properties of EPM-based composites examined by optical microscopy at 130× magnification.

The next part of the following study included the surface properties analysis performed by optical microscopy and the contact angle measurements. Figure 10 shows the images obtained for all tested samples before and after 400 h of thermo-oxidation and climatic aging. As it can be seen, the changes resulting from the thermo-oxidation process are almost invisible, which may mean that the polymer matrix itself exhibits significant resistance to elevated temperatures. However, due to prolonged exposure to atmospheric conditions, the surface of EPM composites was characterized by very small cracks, especially in the case of the ethylene-propylene sample with the addition of quercetin and S.R. 207 dyes.

**Figure 11:**
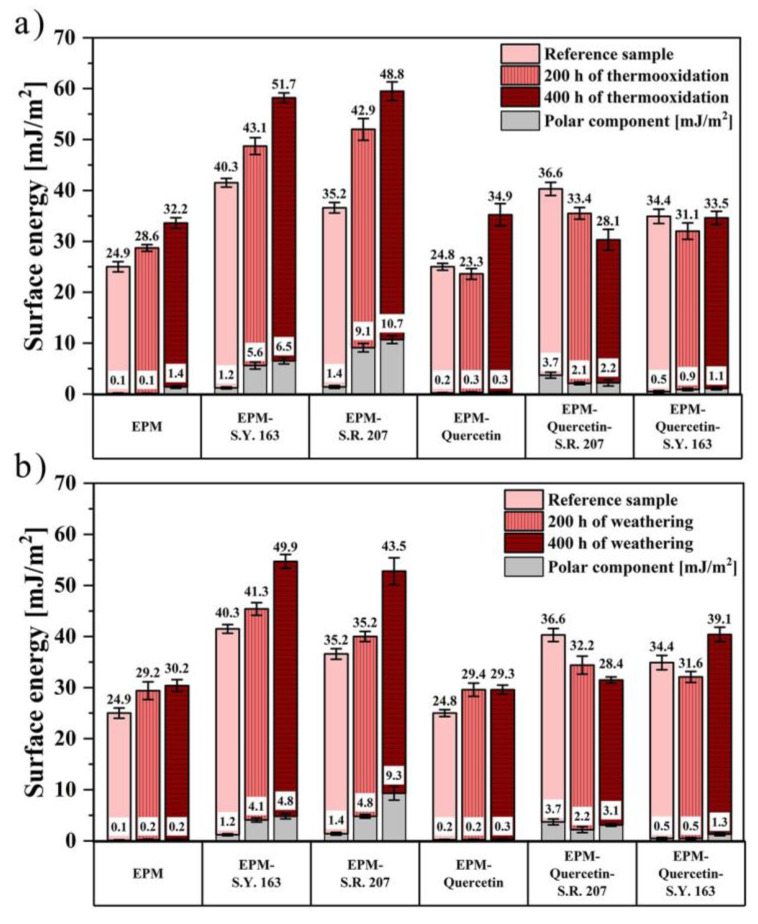
The surface energy of ethylene-propylene materials with specific colorants, calculated by the Owens–Wendt–Rabel–Kealble method before and after 200 and 400 h of (**a**) thermo-oxidation, (**b**) climatic aging.

To better understand the effect of accelerated aging tests and the presence of various colorants on the surface properties of ethylene-propylene rubber, contact angle (CA) measurements were performed and the surface free energy was determined. Wettability is a crucial parameter that defines and limits the application of a material in various industries. The assessment of the wettability can be done by the contact angle measurements and, interestingly, it can be used to assess the surface stabilization of the modified polymer material [60]. Based on the determined contact angle values for the three measuring liquids (distilled water, diiodomethane, and ethylene glycol), the surface free energy (SFE) of ethylene-propylene composites was calculated. The obtained results are shown in Figure 11.

For all samples except for EPM-quercetin-S.R. 207, an increase in the value of surface energy was noticed along with longer exposure to elevated temperature and other atmospheric factors. It can be assumed that they were more susceptible to the degradation process. Moreover, by analyzing the polar components of SFE, ethylene-propylene rubber with the addition of C.I. Solvent Yellow 163 or C.I. Solvent Red 207 dyes is characterized by a significant increase in this constituent due to both thermo-oxidation and climatic aging. This phenomenon can be explained by two main factors, namely the migration of the used synthetic colorants to the EPM surface and degradation of the polymer matrix. The total surface energy is influenced by both the chemical groups present on the material surface and the surface morphology considering the micro/nano scale. The increase in the polar component may be a result of the degradation of very thin layers of the EPM polymer matrix on its surface, and consequently microcracks and holes appear that may reveal dye crystallites located deeper. However, the increase in this component is quite small and does not change the hydrophobic nature of the surface to hydrophilic. On the contrary, the change in surface morphology as a result of aging processes leads to an increase in the surface hydrophobicity of EPM samples with the addition of C.I. Solvent Yellow 163 and C.I. Solvent Red 207 dyes, which was also confirmed by the values of the determined contact angles for water as the measuring liquid. Kim et al. (2006) [61] demonstrated a possibility of using four different dyes, including C.I. Solvent Blue 59 and C.I. Solvent Blue 35, which show super hydrophobicity in the colouring of pure polypropylene fibres. The authors found that the hydrophobicity of the dyes depends on the length of the alkyl substituents, and as it increases, the dyeability of polypropylene fibres significantly improves. Furthermore, Salabert et al. (2015) [62] presented the innovative self-cleaning properties of cotton textiles obtained by adding hydrophobic anthraquinone dyes. Thus, in some cases, a lower wettability and thus higher hydrophobicity can be regarded as an advantage.

On the other hand, the addition of quercetin to composites containing synthetic dye had a significant impact on the values of polar components of surface energy. After 400 h of thermo-oxidation and weathering, the polar component for EPM-quercetin-S.Y. 163 composite was equalled to 1.1 and 1.3 mJ/m^2^, respectively, which is significantly lower than in the case of ethylene-propylene with only C.I. Solvent Yellow 163 dye content. Therefore, this natural flavonoid with antioxidant properties turned out to be effective and had a beneficial effect on slowing down the aging process of the tested materials with the addition of anthraquinone colorants.

#### 2.2.3. Thermal Properties

**Figure 12:**
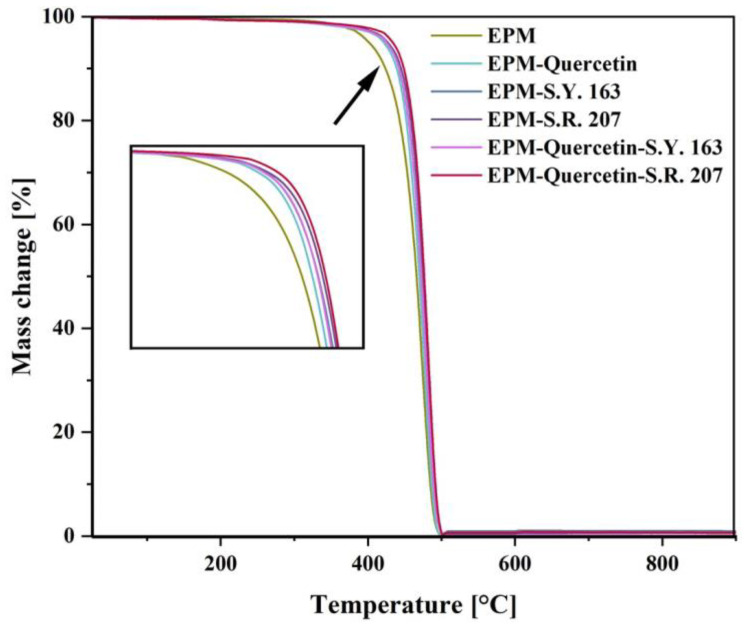
TGA curves obtained for EPM samples with natural and synthetic additives that were heated from 25 to 900 °C.

Thermal analysis plays a key role in assessing the polymer additives stability. Dyes and other used components must be resistant to the polymer processing conditions and further environmental factors. Figure 12 and Table 2 show the thermal stability data for ethylene-propylene rubber with different colorants determined by the thermogravimetric analysis (TGA). As it can be observed, the incorporation of dyes in the amount of 1.5 phr had a significant impact on the thermal stability of the tested composites. For all samples with natural and/or synthetic dye content, a delay in the thermal degradation process has been observed. The excellent thermal resistance of these compounds is generally due to their crystalline structure. In the case of the EPM-quercetin-S.R. 207 sample, the temperature where weight loss was equal to 5% was 433 °C, while for EPM-S.R. 207 material was 427 °C and for pure EPM rubber was 401 °C. This improvement can be related to the nature and position of the substituents presented in these compounds. According to the literature, the better thermal stability of quercetin-containing polymeric materials can be assigned to its chemical structure, especially to the amount of hydroxyl groups [59]. On the other hand, taking into account the obtained values for EPM with anthraquinone dyes (C.I. Solvent Yellow 163 and C.I. Solvent Red 207), the decomposition point of EPM-S.R. 207, which contains a polar benzene ring with a methyl group, is higher than for EPM-S.Y. 163, in which only a polar benzene ring is presented. In addition, in the case of the EPM-quercetin-S.R. 207 composite, its high heat resistance may result not only from the presence of OH groups derived from quercetin, but also from the tendency to form intramolecular hydrogen bonds between quinoid oxides and amino groups present in C.I. Solvent Red 207 as confirmed during FT-IR study.

In the case of quercetin, similar remarks were described by Samper et al. (2012) [63]. In their research, the addition of this natural flavonoid to the polypropylene matrix improved its thermal stability, for which the decomposition onset temperature was equal to 265.9 °C and after quercetin incorporation (0.25%) was 301.1 °C. Another way to improve the thermal stability of coloured polymer products was described by Marzec et al. (2019) [64], who produced ethylene-norbornene films with the addition of an organic-inorganic pigment synthesized by the combination of lawsone dye with an aluminium-magnesium hydroxycarbonate inorganic carrier. Improved thermal behavior, color stability and resistance to solar aging was obtained as a result of strong dye-metal interactions between the lawsone pigment molecules and the LH host. Therefore, the use of different dyestuffs can be a good solution for improving the thermal properties of polymer products.

#### 2.2.4. Fungistatic Tests

It is a widely recognized fact that antibacterial and antifungal properties are crucial in the food packaging industry. They contribute to the prevention of pathogen development in food products, and importantly, they minimize the volume of plastic waste by extending their service life. To provide antibacterial and fungistatic properties of polymeric materials, various additives are introduced into the polymer matrix. Nowadays, more and more often, natural bioactive additives replace traditional chemically active substances applied in the packaging materials. In this research, pure ethylene-propylene rubber, EPM-quercetin-S.Y. 163 and EPM-quercetin-S.R. 207 composites were tested for antifungal activity and the obtained results are summarized in Table 3.

After 24 h of *Candida albicans* contact with the tested materials, the number of yeasts remained at the level comparable to the number of these microorganisms at t = 0, both in the case of the materials with the antimicrobial additive and the reference. This proves the lack of antimicrobial properties of the tested EPM composites. However, no increase in the number of yeasts after 24 h was observed, which indicates fungistatic activity against *Candida albicans* in all materials including the standard (pure EPM sample). No intensive yeast development on the material was observed; the increase was statistically significant over t = 0 but at the same level for all materials.

After 24 h of *Aspergillus niger* contact with the tested ethylene-propylene based samples with dyestuffs addition, the quantity of mold slightly decreased from the initial state (at time t = 0), to a comparable degree, both in the case of the materials with the antimicrobial additive and the reference. It proves the low antimicrobial properties of the tested composites.

## 3. Materials and Methods

### 3.1. Materials

As a polymer matrix, ethylene-propylene rubber (Dutral C0054) supplied by Versalis S.p.A. (Milan, Italy) was used. Dicumyl peroxide (DCP—[C_6_H_5_C(CH_3_)_2_]_2_O_2_) was applied as a crosslinking agent and 1,3,5-triallyl-1,3,5-triazine-2,4,6(1H,3H,5H)-trione (TTT) was used as a crosslinking co-agent. Both were provided by Sigma-Aldrich (Schnelldorf, Germany). A natural colorant—quercetin (quercetin hydrate, ≥95%) was also obtained from Sigma-Aldrich. Two synthetic dyes, C.I. Solvent Red 207 (magenta M6B, C.I.617001) and C.I. Solvent Yellow 163 (yellow GHS, C.I.58840) were delivered by KeyPlast^®^ (Ghent, Belgium) and used as received.

### 3.2. Fabrication of Ethylene-Propylene (EPM) Composites

**Figure 13:**
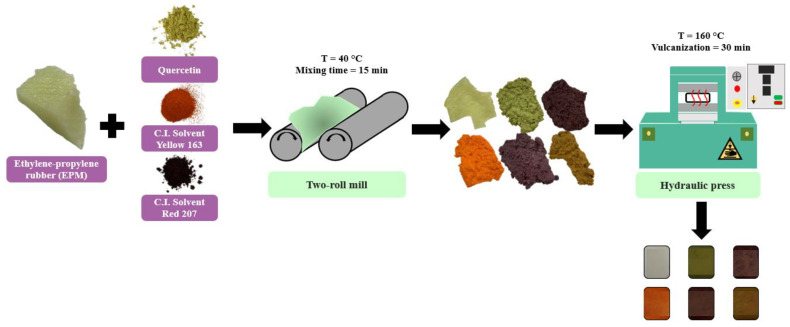
Schematic representation of ethylene-propylene composites fabrication.

Elastomer composites based on ethylene-propylene rubber (EPM) were prepared in an open two-roll mill (roll length and diameter: L = 450 mm and D = 200 mm) at 40 °C. The mixing time was approximately 15 min. First, the EPM rubber was plasticated for 3 min, after which the other ingredients (DCP and TTT) were added. Finally, special selected colorants were implemented to the EPM rubber. Then the prepared mixtures were vulcanized in a hydraulic press at a temperature of 160 °C for 30 min under a pressure of 125 bar. The weight composition of all prepared materials is presented in Table 4, and a simple scheme of EPM composites fabrication is shown in Figure 13.

### 3.3. Controlled Aging Processes

Ethylene-propylene composites were exposed to a weathering test that was performed in compliance with the PN-EN 4892-2 standard “Plastics—Methods of exposure to laboratory light sources—Part 2: Xenon arc lamps”. This research was conducted in a Weather-Ometer Ci 4000 chamber (Atlas Material Testing Technology LLC., Chicago, IL, USA). The operation system consisted of two repeating panels by turns. The first cycle (15 h) was characterized by a temperature of 70 °C and a humidity of 80% with the rainfall turned on. However, during the second panel (10 h), the temperature and humidity were 40 °C and 70%, respectively, with rainfall off. The samples were subjected to weathering for 200 and 400 h.

In addition, the EPM materials were subjected to air at an elevated temperature of 80 °C for 200 and 400 h in a laboratory oven (Binder, Tuttlingen, Germany) with imposed convection. This test was carried out in accordance with PN 82/C-04216 standard “Determination of resistance to accelerated aging in air at increased temperature by means of changes in physical properties”.

### 3.4. Fourier-Transform Infrared and UV-Visible Spectroscopy

Fourier-transform infrared spectroscopy (FT-IR) of colorants powders was performed by using a Nicolet 6700 FT-IR spectrometer from Thermo Fisher Scientific (Waltham, MA, USA). The absorbance spectra were explored within the 3500–500 cm^−1^ range. This research made it possible to define the functional groups presented in the tested samples, with which a radiation interacted. 

UV-Visible spectroscopy of dyes powders was performed by using a UV-Vis spectrophotometer from Thermo Fisher Scientific, Evolution 220 (Waltham, MA, USA). The spectra were recorded at wavelengths of 200 to 900 nm.

### 3.5. Optical Microscopy

The microstructure of EPM composites before and after 400 h of thermo-oxidation and climatic aging was assessed by an optical microscope MZ 6 from Leica Microsystems (Wetzlar, Germany), and the Ts View—digital imaging software was used, which enables the image analysis. This research was performed at room temperature at a magnification of 130×.

### 3.6. Scanning Electron Microscopy (SEM)

The morphology of the colorant powders and EPM composites was assessed based on images received from a scanning electron microscope (Zeiss, ULTRA Plus, Oberkochen, Germany) at a magnification of 1000, 5000 and 50,000× for dyes powders and 5000 and 25,000× for ethylene-propylene materials.

### 3.7. Contact Angle Measurements and Surface Energy Determination

Surface free energy (SFE) of ethylene-propylene samples was determined based on the Owens–Wendt–Rabel–Kealble (OWRK) method by using a goniometer—OCA 15EC from DataPhysics Instruments GmbH (Filderstadt, Germany) and a software SCA 20 (version 1.0). This test consisted in determining the contact angles by applying polar and non-polar liquids (water, ethylene glycol and 1,4-diiodomethane). For each sample, 10 contact angles of each fluid were marked. In general, surface free energy is a sum of polar and dispersive constituents, Equation (1):

(1) $$E_{\mathrm{total}}=E_{\mathrm{polar}}+E_{\mathrm{dispersive}}$$

However, polar and dispersive components were obtained by using the following Equations (2)–(4):

(2) $$\frac{\sigma_{L}\left(1+\cos\Theta \right)}{2\sqrt{\sigma_{L}^{D}}}=\sqrt{\sigma_{S}^{P}}\cdot \sqrt{\frac{\sigma_{L}^{P}}{\sigma_{L}^{D}}}+\sqrt{\sigma_{S}^{D}},\;\;\;\;\;\;\;Y=\mathrm{ax}+b$$

whereas:

(3) $$\;Y=\frac{\gamma_{L}\left(1+\cos\Theta \right)}{2\sqrt{\sigma_{L}^{D}}},\;X=\sqrt{\frac{\sigma_{L}^{P}}{\sigma_{L}^{D}}},\;a=\sqrt{\sigma_{S}^{P}},\;b=\sqrt{\sigma_{S}^{D}}$$

then:

(4) $$E_{\mathrm{polar}}=a^{2}=\sigma_{S}^{P}{\;\mathrm{and}\;E}_{\mathrm{dispersive}}=b^{2}=\sigma_{S}^{D}$$

### 3.8. Color Change Measurements

Color change analysis of ethylene-propylene composites with various dyestuffs was carried out in compliance with the PN-EN ISO 105-J01 standard by using the UV-VIS CM-3600d spectrometer from Konica Minolta Sensing, Inc. (Osaka, Japan) with spectral range of 360 to 740 nm. Each sample was examined at three different points. Then the results were interpreted in the CIE-Lab system, which enables the color description by determining three coordinates. First of which is L—lightness index with a minimum value of 0 (black color) and a maximum value of 100 (diffused white). On the other hand, a positive value of the a* parameter means red, and negative—green. For the third parameter (b*), a positive value indicates yellow color, and negative—blue. The color change of the tested samples was calculated from the following formula, Equation (5) [65]:

(5) $$\mathrm{dE}=\sqrt{{\left(\Delta a\right)}^{2}+{\left(\Delta b\right)}^{2}+{\left(\Delta L\right)}^{2}}$$

Moreover, additional parameters, such as whiteness index (W_i_), chroma (C_ab_), and hue angle (h_ab_) were calculated with the Equations (6)–(8) [27]:

(6) $$W_{i}=100-\sqrt{a^{2}+b^{2}+{\left(100-L\right)}^{2}}$$

(7) $$C_{\mathrm{ab}}=\sqrt{a^{2}+b^{2}}$$

(8) $$h_{\mathrm{ab}}=\{\begin{array}{c} \mathrm{arctg}\left(\frac{b}{a}\right)\;\;\;\mathrm{as}\;\;a>0\wedge b>0\\ 180{}^{\circ}+\mathrm{arctg}\left(\frac{b}{a}\right)\;\;\;\mathrm{as}\;\;\;\left(a<0\wedge b>0\right)\vee (a<0\wedge b<0)\\ 360{}^{\circ}+\mathrm{arctg}\left(\frac{b}{a}\right)\;\;\mathrm{as}\;\;\;a>0\wedge b<0 \end{array}$$

### 3.9. Thermal Analysis

Mass change of the EPM materials as a function of raising temperature was detected by thermogravimetric analysis, which was performed by using a Mettler Toledo^®^ device (Greifensee, Switzerland). These measurements make it possible to determine the initial and maximum temperature of thermal degradation. First, the samples were weighed (6–8 mg) and then heated from 25 to 900 °C under argon atmosphere (heating rate: 10 °C/min).

### 3.10. Fungistatic Activity

This research was conducted based on the ASTM E2180 standard “Test method for determining the activity of incorporated antimicrobial agent(s) in polymeric or hydrophobic materials”. Test strains of the yeast *Candida albicans* ATCC 10231 (Manassas, VA, USA) and the mold *Aspergillus Niger* (syn. *A. brasiliensis*) ATCC 16404 (Manassas, VA, USA) were used in this study. The cultures were stored on slants with Merck’s malt extract agar (MEA, Darmstadt, Germany) medium at 6 °C. The strains were activated before the experiment. The EPM composites were cut into 2 × 2 cm samples. On the tested material surface placed in a sterile vessel, 500 µL of the microorganism suspension in physiological saline with 0.3% agar addition was deposited. Immediately after application (t = 0), the suspension was washed with 5 mL of neutralizer and the number of fungi was determined on the MEA medium by the culture method. The samples with the applied suspensions were incubated at 30 °C for 24 h. After this time (t = 24), the suspension was washed with 5 mL of neutralizer and the number of microorganisms was again determined.

The results are given as the number of units forming colony per 1 cm^2^ of the material surface (jtk/cm^2^). The die rates of microorganisms (D) were determined for the chosen samples with antimicrobial additives (D_B_) and the control material (D_0_) based on the following formula, Equation (9) [66]:

(9) $$D=(\log({\mathrm{number}\;\mathrm{of}\;\mathrm{microorganisms}}_{t=0})-\log({\mathrm{number}\;\mathrm{of}\;\mathrm{microorganisms}}_{t=24}))\;$$

## 4. Conclusions

The aim of this study was to present the influence of bio-based and anthraquinone dyes and their combinations on the optical properties of ethylene-propylene rubber (EPM) after thermo-oxidative and climatic aging. These novel hybrid polymer composites were subjected to thermo-oxidation and weathering for 200 and 400 h, respectively. The first stage of the research involved a detailed characteristics of powder dyes, which included FT-IR, UV-Vis analyses, and scanning electron microscopy. The next phase was the direct addition of quercetin, C.I. Solvent Yellow 163 and C.I. Solvent Red 207 dyes into the EPM polymer matrix. It has been observed that a combination of synthetic and natural colorants can result in better resistance to oxidizing agent and higher thermal stability of EPM composites. Moreover, it has been found that quercetin content strongly affects the color change of ethylene-propylene films. As a result of weathering, EPM-quercetin, EPM-quercetin-S.Y. 163 and EPM-quercetin-S.R. 207 composites changed their coloration towards dark brown shades. Another interesting finding is that these samples exhibited good fungistatic activity against *Aspergillus niger* molds. Therefore, it can be a promising solution for intelligent packaging materials (e.g., in food or pharmaceutical industries), which will inform about the ongoing degradation process through the color change of aging indicators.

## Figures and Tables

**Table 1:** L*, a*, and b* coordinates determined in the CIE-Lab space for all tested samples before and after thermo-oxidation and climatic aging.

Sample	CIE-Lab Space Coordinates [a.u.]					
	Thermo-Oxidation			Weathering		
	0 h	200 h	400 h	0 h	200 h	400 h
L* parameter						
EPM	90.6 ± 0.5	89.7 ± 1.2	87.6 ± 2.2	90.6 ± 0.5	90.6 ± 1.2	89.2 ± 1.5
EPM-Quercetin	47.5 ± 0.4	46.7 ± 0.4	47.8 ± 0.1	47.5 ± 0.4	35.4 ± 0.1	31.7 ± 0.3
EPM-S.Y. 163	56.3 ± 2.3	49.4 ± 0.2	50.2 ± 0.1	56.3 ± 2.3	51.9 ± 0.4	50.6 ± 0.5
EPM-S.R. 207	26.5 ± 0.3	27.0 ± 0.1	27.9 ± 0.3	26.5 ± 0.3	26.7 ± 0.4	27.6 ± 0.2
EPM-Quercetin-S.Y. 163	43.7 ± 0.8	42.6 ± 0.3	41.0 ± 0.3	43.7 ± 0.8	34.7 ± 0.3	33.5 ± 0.5
EPM-Quercetin-S.R. 207	28.3 ± 0.5	27.0 ±0.4	26.5 ± 1.0	28.3 ± 0.5	24.9 ± 0.2	25.7 ± 0.2
a* parameter						
EPM	−1.3 ± 0.1	−2.9 ± 0.4	−1.8 ± 1.0	−1.3 ± 0.1	−2.5 ± 0.1	−2.8 ± 0.2
EPM-Quercetin	−4.7 ± 0.1	−4.9 ± 0.1	−4.4 ± 0.1	−4.7 ± 0.1	3.2 ± 0.1	3.8 ± 0.2
EPM-S.Y. 163	29.8 ± 1.7	34.1 ± 0.2	32.9 ± 1.3	29.8 ± 1.7	33.3 ± 0.3	31.9 ± 1.3
EPM-S.R. 207	9.4 ± 0.8	10.5 ± 1.3	14.2 ± 0.1	9.4 ± 0.8	14.3 ± 0.1	14.8 ± 0.1
EPM-Quercetin-S.Y. 163	12.2 ± 2.5	13.8 ± 0.1	11.8 ± 1.1	12.2 ± 2.5	3.0 ± 0.3	0.6 ± 0.4
EPM-Quercetin-S.R. 207	10.6 ± 0.2	13.9 ± 0.4	14.1 ± 0.3	10.6 ± 0.2	4.4 ± 0.6	7.6 ± 0.5
b* parameter						
EPM	4.2 ± 0.1	10.9 ± 0.3	10.2 ± 1.0	4.2 ± 0.1	7.3 ± 0.3	8.9 ± 0.6
EPM-Quercetin	24.8 ± 1.3	23.3 ± 0.2	23.0 ± 0.1	24.8 ± 1.3	12.5 ± 0.2	7.3 ± 0.1
EPM-S.Y. 163	40.6 ± 2.0	41.7 ± 0.3	40.7 ± 0.3	40.6 ± 2.0	28.7 ± 5.2	36.8 ± 0.2
EPM-S.R. 207	7.9 ± 1.7	7.7 ± 0.7	10.0 ± 0.6	7.9 ± 1.7	9.4 ± 0.9	10.1 ± 0.4
EPM-Quercetin-S.Y. 163	26.8 ± 1.6	27.3 ± 0.4	25.5 ± 0.7	26.8 ± 1.6	10.9 ± 0.2	9.2 ± 0.2
EPM-Quercetin-S.R. 207	9.3 ± 1.2	7.3 ± 0.2	7.1 ± 0.8	9.3 ± 1.2	2.6 ± 0.2	6.6 ± 0.5

**Table 2:** Mass loss temperatures, where T_x%_ is the temperature at which a mass loss is equal to x% (5, 10, 50, and 90%).

Sample	Temperatures of Mass Loss [°C]			
	T_5%_	T_10%_	T_50%_	T_90%_
EPM	401	423	466	484
EPM–Quercetin	421	439	469	486
EPM–S.Y. 163	425	442	473	491
EPM–S.R. 207	427	445	475	491
EPM–Quercetin–S.Y. 163	425	442	472	489
EPM–Quercetin–S.R. 207	433	449	476	491

**Table 3:** Fungistatic activity of the chosen EPM composites containing a combination of synthetic and natural colorants. The values in the table are the average of three measurements (a, b—differences between t = 0 and t = 24; A, B—differences in time of 24 h between samples).

Sample	Number of Microorganisms [jtk/cm^2^]		Log (Number of Microorganisms)		D
	t = 0	t = 24	t = 0	t = 24	
*Candida albicans*					
EPM	5.7 ± 0.64×10^4^	8.3 ± 0.35 × 10^4^	4.76 ± 0.05 ^a^	4.92 ± 0.02 ^bA^	−0.16
EPM-Quercetin–S.Y. 163		9.5 ± 0.42 × 10^4^		4.98 ± 0.02 ^bB^	−0.22
EPM-Quercetin-S.R. 207		7.8 ± 0.53 × 10^4^		4.89 ± 0.03 ^bA^	−0.13
*Aspergillus niger*					
EPM	2.7 ± 0.26 × 10^4^	3.5 ± 0.62 × 10^3^	4.43 ± 0.04 ^a^	3.54 ± 0.08 ^bA^	0.89
EPM-Quercetin–S.Y. 163		6.7 ± 0.35 × 10^3^		3.83 ± 0.02 ^bB^	0.60
EPM-Quercetin-S.R. 207		4.3 ± 0.42 × 10^3^		3.63 ± 0.04 ^bA^	0.80

**Table 4:** Weight composition of ethylene-propylene rubber mixtures with synthetic and natural colorants.

Mixture	Weight Composition (phr)					
	EPM	DCP	TTT	Quercetin	C.I. Solvent Yellow 163	C.I. Solvent Red 207
1	100	4	0.5	-	-	-
2	100	4	0.5	1.5	-	-
3	100	4	0.5	-	1.5	-
4	100	4	0.5	-	-	1.5
5	100	4	0.5	0.7	0.8	-
6	100	4	0.5	0.7	-	0.8

## Data Availability

All the data are available within the article.
